# Influences of High-Level Features, Gaze, and Scene Transitions on the Reliability of BOLD Responses to Natural Movie Stimuli

**DOI:** 10.1371/journal.pone.0161797

**Published:** 2016-08-26

**Authors:** Kun-Han Lu, Shao-Chin Hung, Haiguang Wen, Lauren Marussich, Zhongming Liu

**Affiliations:** 1 Weldon School of Biomedical Engineering, Purdue University, West Lafayette, IN, United States of America; 2 School of Electrical and Computer Engineering, Purdue University, West Lafayette, IN, United States of America; 3 Purdue Institute for Integrative Neuroscience, Purdue University, West Lafayette, IN, United States of America; Penn State University, UNITED STATES

## Abstract

Complex, sustained, dynamic, and naturalistic visual stimulation can evoke distributed brain activities that are highly reproducible within and across individuals. However, the precise origins of such reproducible responses remain incompletely understood. Here, we employed concurrent functional magnetic resonance imaging (fMRI) and eye tracking to investigate the experimental and behavioral factors that influence fMRI activity and its intra- and inter-subject reproducibility during repeated movie stimuli. We found that widely distributed and highly reproducible fMRI responses were attributed primarily to the high-level natural content in the movie. In the absence of such natural content, low-level visual features alone in a spatiotemporally scrambled control stimulus evoked significantly reduced degree and extent of reproducible responses, which were mostly confined to the primary visual cortex (V1). We also found that the varying gaze behavior affected the cortical response at the peripheral part of V1 and in the oculomotor network, with minor effects on the response reproducibility over the extrastriate visual areas. Lastly, scene transitions in the movie stimulus due to film editing partly caused the reproducible fMRI responses at widespread cortical areas, especially along the ventral visual pathway. Therefore, the naturalistic nature of a movie stimulus is necessary for driving highly reliable visual activations. In a movie-stimulation paradigm, scene transitions and individuals’ gaze behavior should be taken as potential confounding factors in order to properly interpret cortical activity that supports natural vision.

## Introduction

Conventional imaging studies in visual neuroscience focus on mapping functional brain regions that process specific visual information such as location, motion, and color [1]. These studies often utilize highly controlled experimental paradigms with artificial or static stimuli, which are, however, too narrowly focused to reflect real-life visual experiences. Naturalistic visual environments are dynamic and complex, containing numerous visual features entangled in space and time [2]. Nevertheless, humans are able to readily and actively explore the surroundings, through neural network activities that give rise to an efficient and effective interplay of visual processing, attention, and behavior (e.g. gaze) [3]. To understand the network basis of this interplay, it is desirable to use natural-vision paradigm for functional neuroimaging, such that brain activity can be recorded and mapped while human subjects are engaging themselves in dynamic and realistic visual environments.

Some recent studies have begun to characterize brain activity during naturalistic movie stimulation [4–6]. Initial findings demonstrate that extended cortical activities are highly reproducible within and between subjects freely watching a commercial movie [5,6]. Depending on the movie stimulus, cortical areas showing reproducible activity may cover the entire visual cortex, as well as attention and oculomotor networks [3]. Such intra- and inter-subject reproducibility has been consistently observed with functional magnetic resonance imaging (fMRI) [5,7], magnetoencephalography [8,9], electrocorticography [10,11], neuronal spike trains and local field potentials [12]. These findings support a central notion that high reproducibility is a robust and perhaps fundamental characteristic of brain activity across a wide range of spatial and temporal scales during naturalistic visual experiences. This notion further allows one to map neural substrates of natural vision by simply assessing the reproducibility of brain activity measured with fMRI [6] or other neuroimaging modalities.

However, the origins of reproducible neural responses to naturalistic visual stimulation are incompletely understood [2]. In particular, it is important to identify and separate the confounding factors that may drive the neural response reproducibility, but bearing little or no relevance to the natural visual content. Specifically, movies possess low-level visual features that are statistically irregular in space and time, and high-level features that drive perception and cognition. To what degree do the high- or low-level features drive the reproducible brain response remains elusive. Russ and Leopold have shown that reproducibility in cortical activity is explained by both low-level (e.g. contrast and luminance) and high-level features (e.g. faces, animals, and biological motion) [2]. Hasson et al. have shown that disrupting the temporal structure of a movie would reduce the reproducibility of cortical responses [13]. As such, the high-level features resulting from integrating information in time may drive, at least in part, the reliable cortical activity. However, it is unknown whether and where cortical response reproducibility may still remain, in the absence of any high-level features when both spatial and temporal structures are disrupted to render a movie stimulus perceptually meaningless.

In addition, commercial movies typically contain instantaneous scene transitions due to film editing [14]. The scene transitions may appear as transient stimulus events that may induce reproducible responses while being unrelated or loosely related to the natural context. Moreover, when subjects are freely engaged to a movie stimulus, they may receive inconsistent retinal inputs that depend on their individual gaze behaviors [3]. The effects of the varying gaze behavior on brain activity and its reproducibility also remains to be addressed.

In this study, we aimed to address these questions by using data collected with concurrent fMRI and eye tracking from human subjects receiving repeated movie stimuli. Fourier shuffling was applied to the movie to create a spatiotemporally scrambled control stimulus such that the low-level visual features were dissociated from the high-level natural content in the intact movie. By comparing the reproducibility in fMRI activity during the scrambled vs. intact stimuli, we attempted to disentangle the individual contributions of the low and high-level features to the reproducibility of the movie evoked response. We also assessed the contributions of individuals’ gaze behavior to fMRI activity and its reproducibility within and across subjects, and further evaluated the extent to which the reproducibility in fMRI activity was confounded by scene transitions in the movie. Results obtained with the above analyses are expected to help refine the design, analysis, and interpretation of present and future natural vision imaging studies.

## Materials and Methods

### Subjects

Sixteen human subjects (10 women, ages 20−31) participated in this study in accordance with a research protocol approved by the Institutional Review Board at Purdue University. All subjects were healthy volunteers with normal vision. Informed written consent was obtained from every subject. Among the 16 subjects, three subjects’ data were excluded from the subsequent analyses either because they fell asleep with eyes closed or had excessive head movement.

### Stimuli

**Fig 1:**
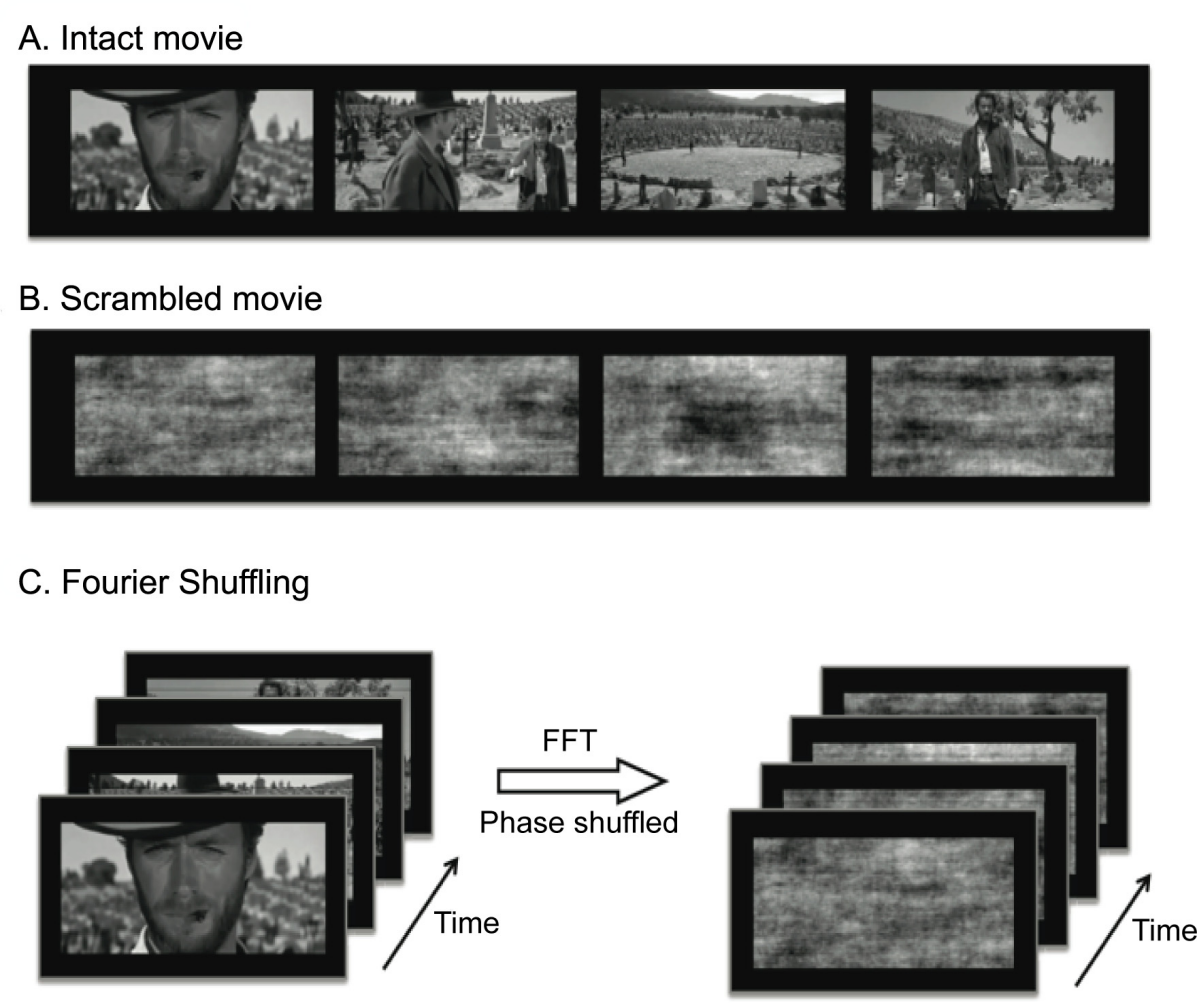
Illustration of the naturalistic vs. scrambled visual stimuli. Example frames from the intact movie (A) and the scrambled movie (B). The scrambled movie was created by shuffling the phase of the intact movie in the frequency domain (C).

An uninterrupted segment of movie (5:37 minutes in length, from 163:06 to 168:50 minutes in The Good, the Bad, and the Ugly, directed by Sergio Leone) was converted to gray scale to provide a naturalistic visual stimulus. From this movie, a scrambled movie was further created to keep the same statistical distribution of pixel intensity as the intact movie, yet rendering itself perceptually meaningless. Specifically, we used 3D Fast Fourier Transform (FFT), as implemented by the function ‘fftn’ in MATLAB, to convert the intact movie into the frequency domain, in which the phase was randomly shuffled while the magnitude remained unchanged; then inverse FFT was applied to the phase-shuffled data to generate a scrambled version of the original movie. As illustrated in Fig 1, the intact and scrambled movies were matched in low visual features, whereas the contrast between them was specific to the presence vs. absence of high-level natural content independent of low-level visual properties.

### Paradigm

Among the 13 subjects, seven underwent four sessions of fMRI acquisition with visual stimulation: two sessions for the intact movie, and two sessions for the scrambled movie. These subjects were instructed to freely view the movie stimuli. Four subjects underwent two repeated fMRI sessions for the intact movie stimulation, while they were instructed to fixate at a cross-hair (0.8 × 0.8 degrees in width and height) at the screen center. Two subjects underwent eight sessions of fMRI acquisition: two sessions for the intact movie (free-viewing), two sessions for the intact movie (fixation), two sessions for the scrambled movie (free-viewing), and two sessions for the scrambled movie (fixation).

Every stimulation session started with a blank gray screen presented for 42 seconds, followed by the intact or scrambled movie presented for 5 minutes and 37 seconds, ended with the blank screen again for 30 seconds. No sound was played during the movie. The order of the above stimulation sessions was randomized and counterbalanced across subjects.

### MRI

All experiments were conducted in a 3T MRI system (Signa HDx, General Electric, Milwaukee). A 16-channel receive-only surface phase-array coil (NOVA Medical, Wilmington) was used throughout every experiment. T_1_-weighted anatomical images were acquired with a spoiled gradient recalled acquisition (SPGR) sequence (256 sagittal slices with 1 mm thickness and 1×1 mm^2^ in-plane resolution, TR/TE = 5.7/2ms, flip angle: 12°). FMRI data were acquired with the standard single-shot, gradient-recalled (GRE) echo-planar imaging (EPI) sequence (38 interleaved axial slices with 3.5 mm thickness and 3.5 × 3.5 mm^2^ in-plane resolution, TR = 2000ms, TE = 35ms, flip angle = 78°, field of view = 22 × 22 cm^2^, in-plane acceleration by a factor of two based on sensitivity encoding (SENSE), no acceleration in the slice direction).

### Eye-Tracking

The visual input was presented using the MATLAB-based Psychophysics Toolbox [15,16], and they were delivered to the subjects through a binocular goggle system (NordicNeuroLab, Norway) mounted on the head coil. The display resolution was 800 × 600. An MR-compatible monocular eye-tracking camera was integrated into the goggle system to monitor the left eye under infrared illumination. The eye-camera data were transmitted to an eye-tracking system (ViewPoint, Arrington Research, USA), which captured the eye movement and tracked the subject’s gaze location at 30 Hz during the stimulation session. To ensure reliable and accurate eye tracking, the system was recalibrated prior to every stimulation session. Through the goggle system, the visual field covered by the movie was about 26.9-by-20.3 degrees. When the subjects were instructed to fixate at the screen center, the fixation was not ensured by any fixation task, but confirmed retrospectively with eye-tracking data.

### Pre-processing

MRI and fMRI data were preprocessed by using FSL [17] and AFNI [18]. Briefly, T_1_-weighted anatomical images were non-linearly registered to the MNI brain template. T_2_*-weighted functional image series were corrected for slice timing, registered to the first volume within each series to account for head motion, masked out non-brain tissues, aligned to the T_1_-weighted structural MRI, registered to the MNI template and resampled into 3×3×3mm^3^ voxels. After discarding the first six volumes, the fMRI data were temporally de-trended by using a third-order polynomial function to model the slow signal drift, and spatially smoothed by using a 3-D Gaussian filter with 6mm full width at half maximum (FWHM).

As the focus of this study was on brain activity during sustained and dynamic movie stimuli, we only used the data from 12 seconds into the movie to the end of the movie. Data from other periods were all excluded from subsequent analyses to avoid the transient fMRI response shortly after the gray screen was replaced by the movie presentation.

### Inter- and intra-subject reproducibility in fMRI activity

As previously published elsewhere [5], the map of visually evoked activations was obtained by assessing the zero-lag Pearson’s cross correlation in every voxel’s time series between two repeated presentations of the identical movie for the same or different subjects. Specifically, the intra-subject correlation was measured for every voxel and each subject, whereas the inter-subject correlation was measured for every voxel and each pair of different subjects. The intra- and inter-subject correlation coefficients were converted to z scores through the Fisher’s r-to-z transform with the degree of freedom set as the number of time points minus one.

Further, the intra- or inter-subject reproducibility was tested for statistical significance in the group level. The intra- or inter-subject reproducibility and its significance were evaluated separately for the intact or scrambled movie in a free-viewing or fixation condition. For the intra-subject reproducibility, the z-transformed correlation coefficient (or z score) between sessions from the same subject was averaged across subjects; the voxel-wise statistical significance was evaluated by using one-sample t-test, with p < 0.0033, DOF = 8, multiple comparison corrected by controlling the false discovery rate (FDR) < 0.03 [19] (FDR is also denoted as *q* in this paper). Note that the same method for the correction for multiple comparison was used for all other statistical tests in the study, unless it was described otherwise. For the inter-subject reproducibility, the voxel-wise cross correlation was obtained for each pair of different subjects. The pair-wise correlations were not all independent because each subject could be involved in more than one subject pair. This lack of independence invalidated the use of parametric statistical tests, such as the above-mentioned one-sample t test. Instead, we used a non-parametric resampling based statistical inference as implemented in ISC-toolbox (www.nitrc.org/projects/isc-toolbox/) and described in details elsewhere [20]. Briefly, an empirical distribution was obtained for inter-subject cross-correlations given the null hypothesis that such correlations were trivial and non-significant. To do so, every subject’s signals were circularly shifted in time by a random and different amount so that the signals from different subjects were no longer aligned in time. Following the temporal resampling, cross correlations were computed between the resampled time series at the same or different voxels from different subjects, effectively yielding a null resampling distribution with 10 million samples of trivial inter-subject cross correlations. This resampling-based statistical inference also took into account the auto-correlated nature of the fMRI signal. Without resampling, all inter-subject cross correlations were tested against this empirical null distribution, yielding the p values for significance test, corrected for multiple comparison with FDR < 0.03.

Note that either physiological (respiratory or cardiac) fluctuations or head motion parameters were not regressed out from the fMRI signals prior to the calculation of the intra- and inter-subject reproducibility, because such task-unrelated fluctuations were not expected to contribute to the cross correlation between signals from different fMRI sessions.

### Assessing the effects of gaze behavior

In the natural vision paradigm, the interpretation of reproducible fMRI activity in terms of fluctuating visual features may be of concern due to the presence of multiple confounding factors that may contribute to the fMRI signal, and drive or disrupt the signal reproducibility. We first addressed the degree to which fMRI activity and its reliability could be accounted for by the subjects’ varying gaze behavior monitored with eye tracking.

Specifically, every subject’s gaze locations were estimated from instantaneously captured eye images. The time series of the horizontal (x) and vertical (y) variations were first filtered by moving average within a 1s window, in order to minimize the influence of the extreme values caused by eye blinking. After removing the mean from the x and y time series, we characterized the gaze behavior by calculating the instantaneous saccadic amplitude (in degrees), which was the spanning visual angle between two consecutive gaze locations based on a gaze displacement model, as described elsewhere [21,22]. The saccadic amplitude signal was further demeaned, and was convolved with the canonical hemodynamic response function (HRF), as implemented in SPM (www.fil.ion.ucl.ac.uk/spm/). The result were further resampled to match the fMRI sampling times after applying an anti-aliasing low-pass filter with the cutoff frequency at 0.25 Hz. As such, a single regressor was derived from the time-varying saccade amplitude as an explanatory variable for the fMRI signal.

The zero-lag cross correlation between the fMRI signal and the saccade-amplitude regressor was evaluated for every voxel. The correlation coefficients were transformed to the z scores, and tested across subjects for significance by using one-sample t-test (p < 0.011, DOF = 17, FDR < 0.03). To assess the gaze effects on the reproducibility of fMRI activity, the gaze-related regressors were excluded from each voxel time series by using a linear nuisance variable regression (NVR). The difference in the intra-subject reproducibility with vs. without the NVR was tested for significance by using paired t-test (p < 0.03, DOF = 8, uncorrected).

Alternatively, the effects of gaze behavior were addressed by comparing the intra-subject reproducibility of fMRI activity when the subjects were freely watching the movie (n = 9) versus watching the same movie but with their eyes fixated (n = 6). The latter served as a control experiment without gaze behavioral variation either within or across subjects. Between these two conditions, the voxel-wise difference in the z-transformed intra-subject correlation was tested for statistical significance by using two-sample t-test (p < 0.03, DOF = 13, uncorrected).

### Assessing the effects of scene transitions

We further addressed the effects of scene transitions due to film editing. The scene transitions manifested themselves as transient and discontinuous changes in visual input. The occurrence times of scene transitions were first detected if the sum of the absolute pixel difference between two consecutive movie frames exceeded a given threshold, and then visually affirmed. A train of binary events was defined as a time series of ones and zeroes, with one indicating the occurrence of a transition event. The binary events were further weighted by the estimated motion between two consecutive movie frames at the transition time based on a block-matching algorithm implemented as a built-in function (vision.BlockMatcher) in MATLAB (the block size = 9) [23]. It measures the movement fields in pixel blocks between two images, regardless of the perceptual meanings of these images. The motion-weighted transition events was further convolved with the HRF, and then filtered and resampled to construct a regressor for the fMRI signal. We used this regressor to evaluate the effects of scene transitions on fMRI activity (p < 0.0017, DOF = 17, FDR < 0.03) and its reproducibility (p < 0.001, DOF = 8, FDR < 0.03) using the similar correlation and regression analyses as used for addressing the effects of gaze behavior.

While the above analyses were done with the volumetric data, the results were displayed on both the MNI volume template and the cortical surface templates [24].

## Results

### FMRI reproducibility reveals visually evoked activations

We used a long, continuous, dynamic, and complex movie to investigate the neural substrates underlying natural vision. For such a stimulus paradigm, it was difficult to define accurate response predictors required for the conventional fMRI analysis. Using the method proposed by Hasson et al. [5], we localized the visually evoked responses to areas where the fMRI time series was cross correlated between two repeated presentations of the identical movie for the same (intra-subject) or different (inter-subject) subjects. The intra- and inter-subject reproducibility in fMRI activity was statistically evaluated in both individual and group levels to yield the activation maps induced by the movie stimulation.

**Fig 2:**
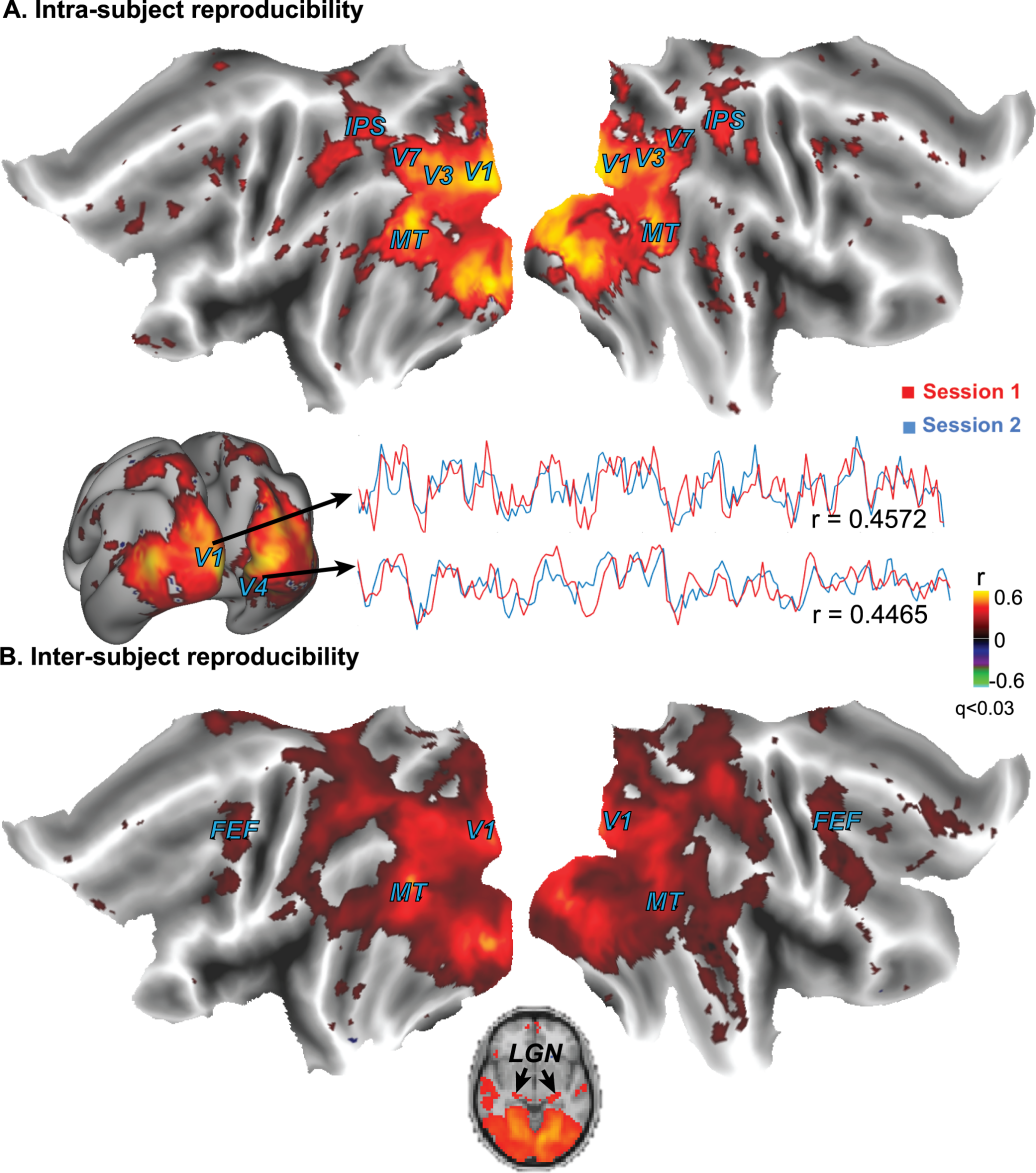
**Brain activations with the intact movie were found at regions that showed significant intra-subject (A) and inter-subject (B) correlations in cortical activity during free movie watching.** The mapping results were based on data from nine subjects. From a single subject, the fMRI signals from two voxels within the primary visual cortex (V1) and the lateral occipito-temporal gyrus (V4) are shown as examples to illustrate the intra-subject reproducibility in cortical activity. The color indicates the cross correlation.

The intact movie gave rise to significant intra- and inter-subject reproducibility in fMRI activity at extended occipital areas that covered almost the entire visual cortex (Fig 2). The reproducibility was stronger within subjects (Fig 2A) than across subjects (Fig 2B), whereas the inter-subject reproducibility was significant even at lateral geniculate nuclei (LGN), and also extended to the parietal and frontal lobes (Fig 2B). These results corroborate the previous finding that naturalistic visual stimulation induces highly reproducible and widely extended cortical activity [5,6], and further extend this finding by showing reproducible thalamic responses during free watching of a movie.

### Spatiotemporal scrambling eliminates reproducible brain activity

**Fig 3:**
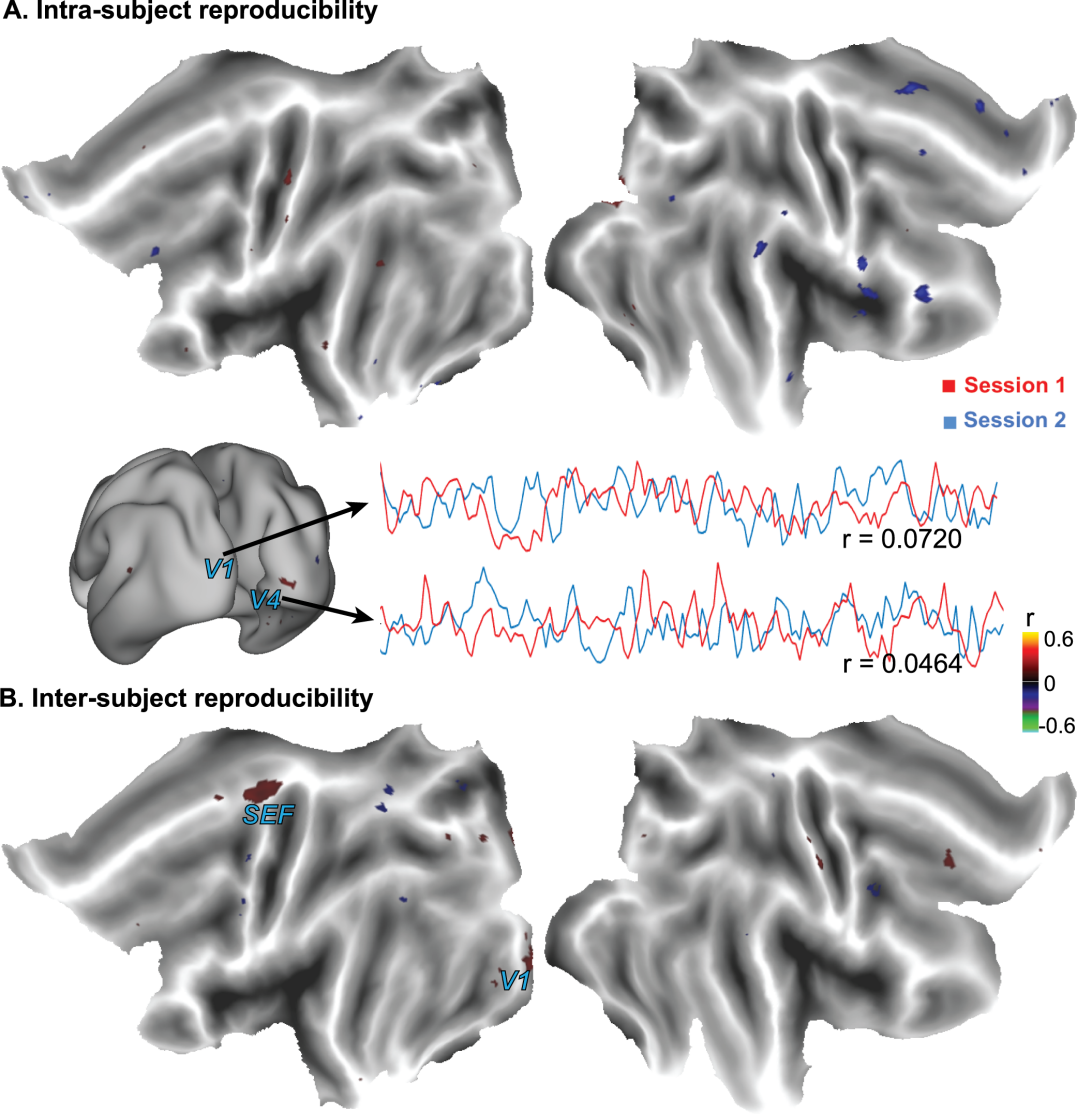
**Cortical activations with the scrambled movie were reduced and confined to V1, as revealed by the intra-subject (A) and inter-subject (B) reproducibility of the fMRI signal.** The mapping results were based on data from nine subjects. From a single subject, the fMRI signals from two voxels within the primary visual cortex (V1) and the lateral occipito-temporal gyrus (V4) are shown as examples to illustrate the relatively low intra-subject reproducibility in cortical activity. The color indicates the cross correlation.

Next we asked whether highly reproducible cortical activity was unique to natural vision, or it might also result from irregularly fluctuating visual stimulation that carried no naturalistic content in every movie frame. To address this question, we scrambled the movie to render it perceptually meaningless while preserving low-level visual features, such as luminance and spatial/temporal frequency. When subjects freely watched the scrambled movie, the intra-subject reproducibility of the fMRI signal was not significant anywhere in the brain (Fig 3A), while the inter-subject reproducibility was barely significant only within the primary visual cortex (V1) (Fig 3B).

**Fig 4:**
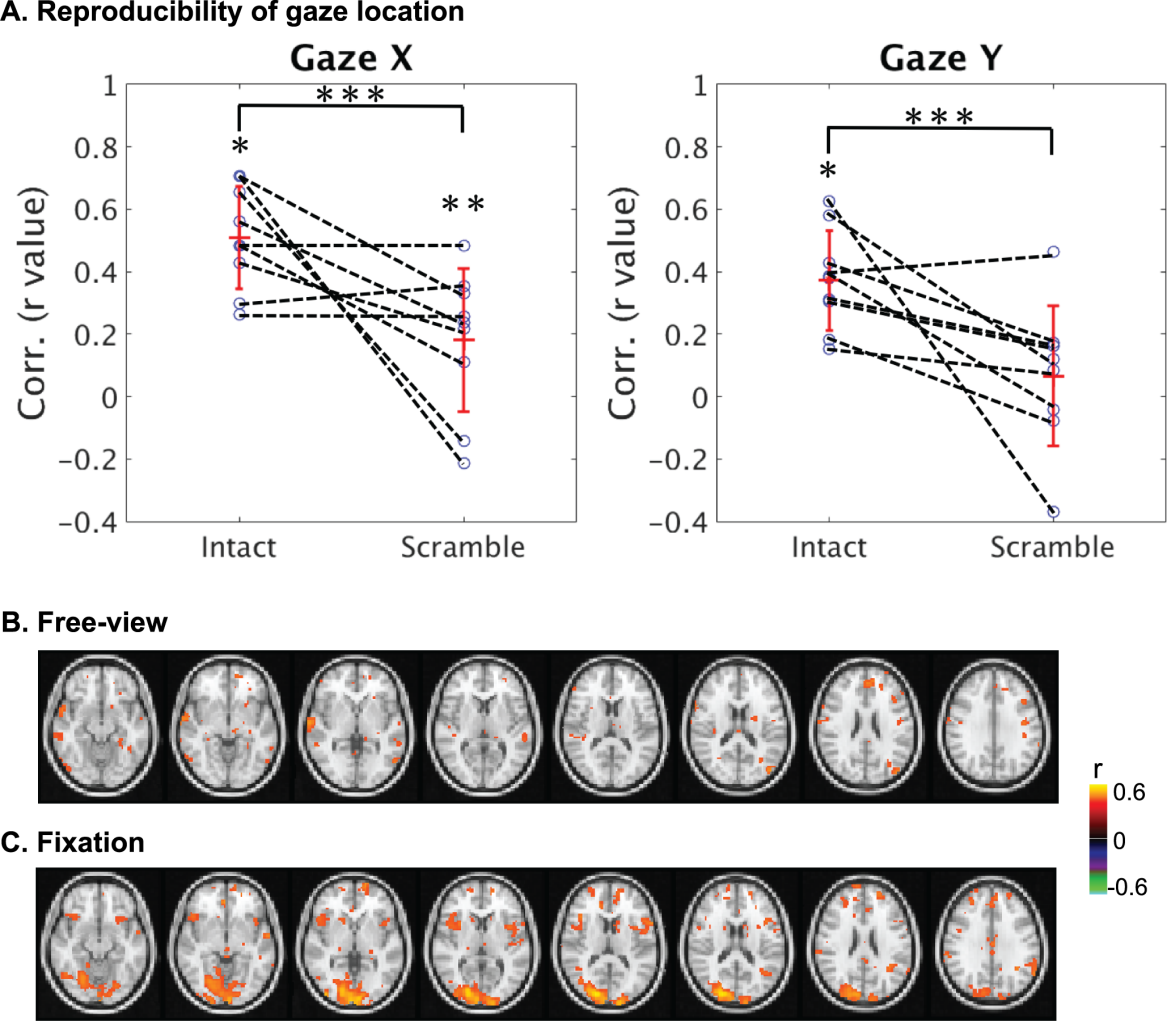
Effects of varying gaze behavior on cortical reliability when watching the scrambled movie. (A) For most subjects (except one), the horizontal and vertical variations of the gaze location were more consistent for the intact (non-scrambled) movie than for the scrambled movie. Error bars represent the standard deviation across subjects. (B) For an example subject, when the subject did not fixate at the screen center, there was a lack of reliable responses (C) The scrambled movie induced reliable responses within V1 when they fixated at the screen center. In both (B) and (C), the color indicates the intra-subject cross correlation in voxel time series (*: p < 0.0005, **: p < 0.05, ***: p < 0.03). These results were based on eye-tracking data from nine subjects who freely watched both the intact and scrambled movies.

We further questioned whether the lack of reproducible activity during the scrambled movie might be attributed to variable gaze trajectories within and across subjects. The absence of natural content could affect the subjects’ involuntary attention and cause different gaze patterns during the two repetitions of the scrambled movie. Indeed, the intra-subject cross correlation in the gaze trajectory was significantly less (p<0.03) for the scrambled movie than for the intact movie (Fig 4A). However, the variable gaze pattern could not entirely account for the reduced degree and extent of reliable cortical responses, because the reproducibility in the fMRI signal was confined to V1 even when eyes were fixated at the screen center (Fig 4C) compared to the free-viewing condition (Fig 4B).

Therefore, highly reproducible and widely extended fMRI response to a continuous, complex, and dynamic visual stimulus requires the stimulus to be naturalistic, whereas a non-natural stimulus with matched low-level visual properties induces a much less degree and extent of time-locked and reproducible cortical activity.

### Gaze behavior affects oculomotor activity during natural vision

**Fig 5:**
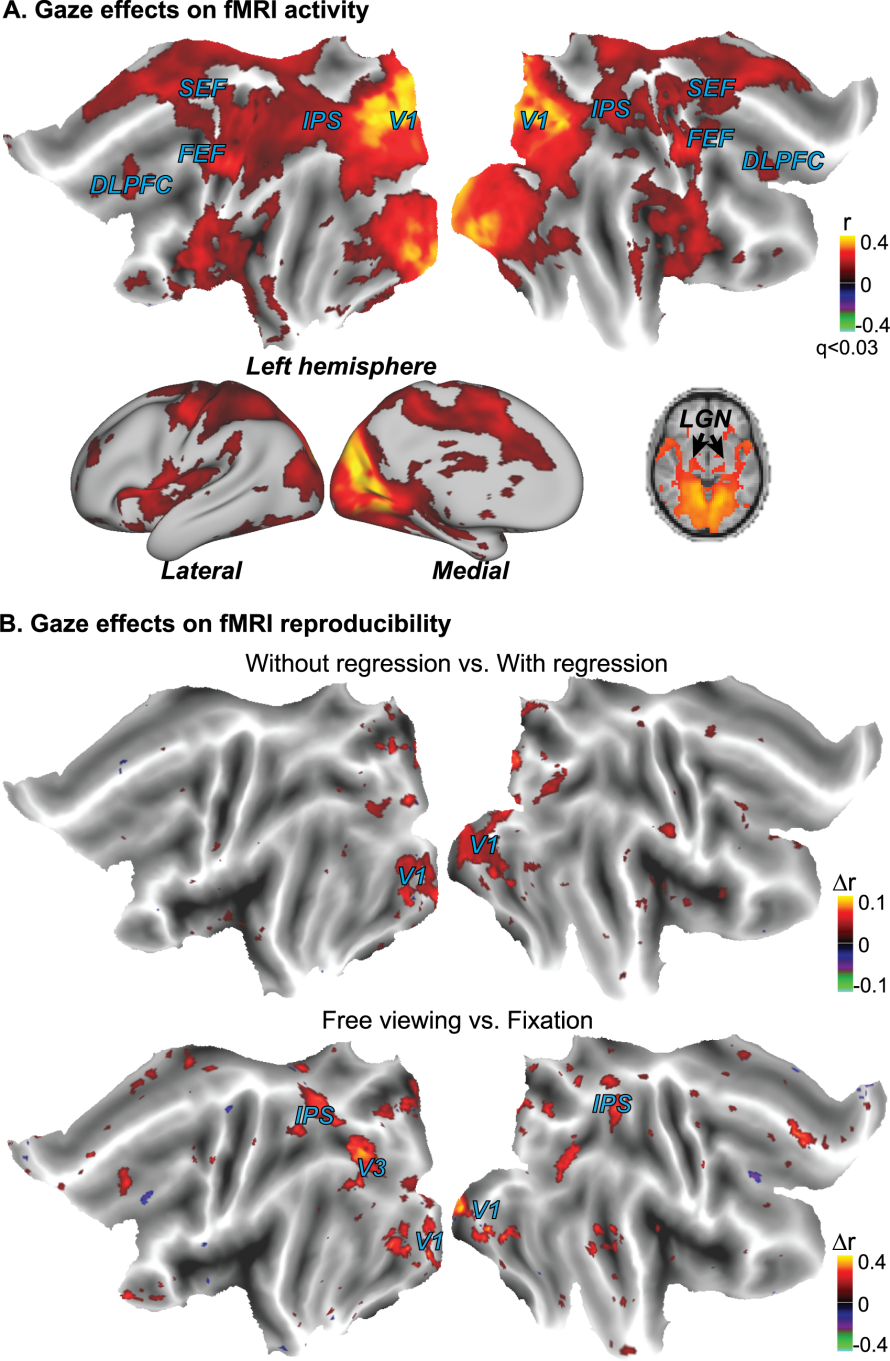
Varying gaze behavior contributed to fMRI activity and its reproducibility during natural movie stimulation. (A) Cross correlation between the fMRI signal and the time-varying saccade amplitude during free movie viewing (p < 0.011, FDR < 0.03). (B) The upper panel shows the difference (without regression minus with regression) in intra-subject correlation when taking the four gaze behaviors as the nuisance variables (p < 0.03, uncorrected). The lower panel shows the difference in intra-subject correlation when taking free-viewing the intact movie as the experimental group (n = 9) while taking the eyes-fixated movie watching (n = 6) as the control group (p < 0.03, uncorrected). The color shows the difference in cross correlation following the t-test.

The changing scene content in a naturalistic movie may drive varying gaze behavior as monitored with eye tracking. The varying gaze location, in turn, may alter the retinal input and thus the induced cortical activity. To assess the dependence of cortical fMRI reliability on gaze behavior during natural vision, we measured the zero-lag cross correlation between the fMRI signal and the fluctuation of the saccade amplitude derived from instantaneous eye movements. We found that the saccade-amplitude fluctuation was significantly correlated with the fMRI activity notably in the part of the V1 that represented the peripheral visual field, as well as in other cortical areas along both dorsal and ventral streams, including intra-parietal sulcus (IPS), frontal eye fields (FEF), supplementary eye fields (SEF), dorsal-lateral prefrontal cortex (DLPFC), and insula (Fig 5A).

The gaze behavior was found to vary not only across sessions and subjects (Fig 4A), but also within a session, being more consistent for movie frames showing salient objects or human faces. This variation could affect the level and extent of activity reproducibility when the subjects were freely watching the movie. To assess this effect, we compared the intra-subject reproducibility in the fMRI signal with and without regressing out the individual variation in gaze movement. The contrast (without regression minus with regression) was significant primarily in peripheral V1 (Fig 5B, top panel). To further address the gaze-related effects, we compared the intra-subject reproducibility in fMRI signals between the free-viewing (n = 9) and fixation conditions (n = 6), while the gaze behavior varied within and across subjects in the former condition but not in the latter. The contrast map (free-viewing minus fixation) between these two conditions showed localized gaze-related effects, qualitatively similar to those observed with the nuisance variable regression (Fig 5B, bottom panel); but it revealed more areas, including IPS and DLPFC, in addition to peripheral V1.

Therefore, When free viewing a movie, the gaze behavior affects brain activity primarily in peripheral V1, as well as in cortical regions likely involved in controlling or responding to saccades [25,26]. However, the gaze behavior does not significantly confound or explain widely distributed cortical activations reliably induced by the naturalistic stimulation.

### Scene-transition events induce reliable responses in visual cortex

**Fig 6:**
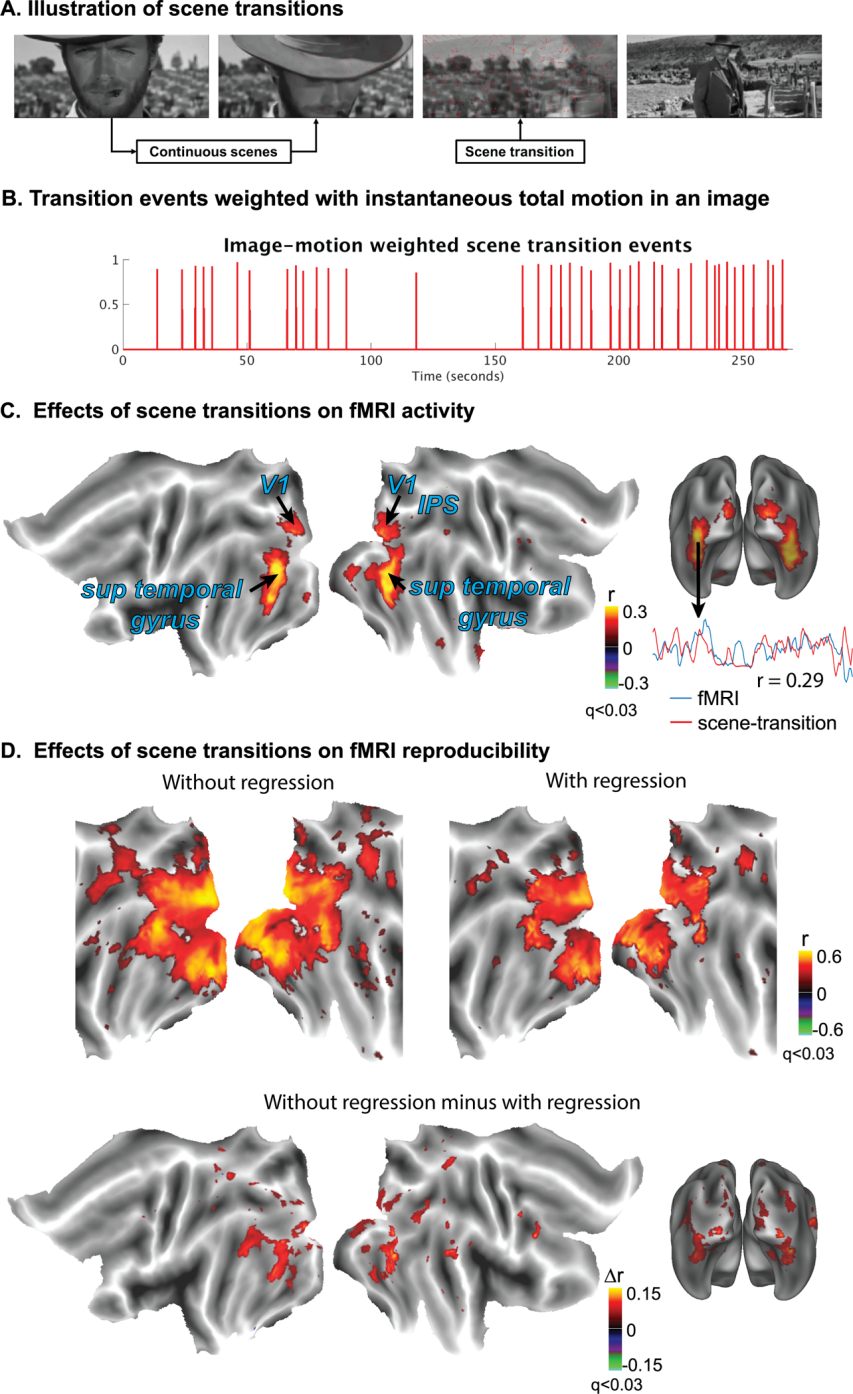
Dependence of cortical fMRI activity and its reproducibility on scene transitions during natural vision. (A) A scene transition (video shot boundary) occurred as visual input changed abruptly due to film editing. (B) A train of scene-transition events were defined by the timing and degree of the transition evaluated by the total motion change between two adjacent movie frames. (C) Cross correlations were calculated between the fMRI signals and the regressor derived from the scene-transition events (p < 0.0017, DOF = 17, FDR < 0.03). Representative time courses from one subject are shown to demonstrate the similarity between the scene-transition predicted response and the fMRI signal from a voxel in the ventral visual pathway (D) Comparison between the intra-subject reproducibility without (left) vs. with (right) regressing out the scene-transition induced response. Their difference (bottom) was evaluated and shown to depict the effects of scene transitions on the reproducibility of cortical activity during natural vision. The color shows the correlation coefficient or the difference in correlation coefficient, following the t-test (p < 0.001, FDR < 0.03).

Next, we examined to what degree brain activity was driven by scene transitions in the movie, which reflected the transient change in visual input (Fig 6A). The scene transitions were found to contribute to cortical responses mostly at areas along the ventral visual stream (Fig 6C), where the fMRI signal was significantly correlated with a modeled response based solely on a train of discrete scene-transition events weighted by the total motion between consecutive movie frames at each transition time (Fig 6B). In addition, these scene transitions also confounded the intra-subject reproducibility in cortical fMRI activity during the movie. Regressing out the contributions from scene transitions to the fMRI signal significantly reduced (p < 0.001, FDR < 0.03), but did not eliminate, the reproducibility of cortical activity at distributed extrastriate cortical areas, including those along the ventral pathway (Fig 6D).

Therefore, the scene transitions in commercial movies partly drive the extrastriate cortical activity, confounding the assessment and interpretation of the reproducibility in fMRI activity during naturalistic visual stimulation.

## Discussion

In this study, we used concurrent fMRI and eye tracking to assess the degree and extent to which reproducible brain activity could be affected by various experimental and behavioral factors during free viewing of sustained, dynamic, and complex movie stimuli. Results demonstrate that the varying gaze behavior affected the reproducibility of fMRI activity in the peripheral V1, and that the scene transitions in the movie affected the reproducibility in the areas along the ventral visual pathway. For these areas, mathematically removing the confounding contributions of these factors from fMRI signals led to significantly less (but not diminished) reproducibility in cortical activity. In addition, we found that the highly reliable and widely extended activations evoked by a movie was mainly due to the naturalistic content of the movie. Removing the perceptually meaningful content while keeping the low-level visual properties largely reduced the degree and extent of the evoked cortical response. Taken together, measuring and mapping the reproducibility of fMRI activity constitutes a robust functional imaging technique to uncover neural processes responsible for visual processing and perception in a dynamic, complex, and realistic context. However, it is worth taking into account (more importantly) scene transitions and (less importantly) individuals’ gaze behavior as confounding factors.

### Reliable visually evoked activations arise from naturalistic content

In the seminal work by Hasson et al., an important finding was the functional selectivity of cortical activity during natural movie stimuli [5]. For example, face or building related regions exhibited dynamic cortical fluctuations with peak activity selectively occurring when various forms of face or building appeared in a movie (see Fig 3 in Hasson et al., 2004). This finding pointed to a possibly exciting opportunity to infer natural and abstract visual content from dynamic cortical activity of the brain in action [6]. However, natural vision paradigms with movie stimuli are different from conventional fMRI paradigms in that fluctuations and patterns of cortical activity are not analyzed by contrasting the target vs. control conditions. The lack of a control condition requires more systematic investigations on various sources that modulate cortical activity, in order to disentangle the sources of interest for visual perception (e.g. house, building) as well as those unrelated or loosely related to perception (e.g. scene transitions, eye movements).

Here, we asked whether trivial movie stimuli in the absence of perceptually meaningful content would also induce reliable cortical activity to confound the interpretation of activity fluctuations in terms of whether, what, and how natural information is processed and perceived by human subjects. Previous studies have reported that individual cortical neurons could fire reliably with precise spike timing given irregularly fluctuating or transient inputs, but not with constant or steady state stimuli [27,28]. These findings suggest the intrinsic reliability and precision of neural coding may depend on the statistical irregularity (or complexity) of the input information [27]. Extending this coding characteristic from single neurons to distributed cortical networks, a valid alternative hypothesis is that reliable cortical activity may be primarily attributed to the irregular visual patterns of the movie stimulus, as opposed to its naturalistic nature. Although natural vision necessarily entails irregular and dynamic visual patterns, spatiotemporally complex visual inputs do not always convey natural content. Therefore, it is unclear whether and to what extent the naturalistic nature of the visual stimulus is necessary for the highly reliable and widely distributed neural responses during sustained, complex and dynamic visual stimuli.

To address this question, we separated the irregular vs. naturalistic property of a complex movie stimulus by scrambling the naturalistic movie stimulus to dissociate the low-level irregular features from the high-level naturalistic features (Fig 1). Results indicate that the evoked cortical activations, in terms of intra- and inter-subject reproducibility of fMRI activity, were largely eliminated by the absence of the natural content when subjects freely watched the scrambled movie, with barely significant residual activations mostly confined to V1 (Fig 3). This finding suggests that the reliable cortical activation with natural vision mainly results from the natural content in the movie, rather than its spatiotemporal irregularity and complexity.

In real life, the natural content is embedded into spatial and temporal structures of visual elements. Disruption in either or both of these structures compromises, to a varying degree, the natural information. In previous studies, researchers scrambled movie frames in time to disrupt the temporal structure within a varying period [13]. When the temporal structure was disrupted, early visual areas still exhibited highly reliable responses, whereas the response reliability at higher visual areas was reduced to a varying degree depending on the length of the time-scrambled window [13]. This important finding has led to the discovery of a hierarchical organization of the temporal receptive field, similar to that of the spatial receptive field [13]. In line with (but different from) these studies, we scrambled the visual elements not only in time but also in space by using phase-shuffling. Such a scrambled movie disrupted both spatial and temporal structures of a natural movie stimulus, such that movie frames did not appear natural either individually or collectively. This complete absence of naturalistic information was different from time-scrambled movies, for which natural content was still preserved in individual movie frames, or connected in a longer (non-scrambled) time scale. In the present study, the lack of reliable cortical response to spatiotemporally scrambled movie stimuli extends the previous findings, and further indicates the importance of the natural content in driving highly reproducible and widely extended brain activity under movie stimulation.

It might be expected that the scrambled movie did not evoke reliable cortical responses at higher visual areas, as these areas have been conventionally considered to be responsive to high-level visual features. However, the lack of reliable responses to the scrambled movie even at early visual areas came as a surprise. Neurons in lower visual areas (e.g. V1, V2) have small receptive fields and are considered to respond to low-level features of stimuli [29], whereas neurons in higher cortical areas are responsible for increasingly complex and abstract features [30,31]. Neuronal connections to early visual areas arise in part from retinal inputs through bottom-up or feed-forward pathways, and also in part from higher visual areas through top-down or feedback pathways. Both bottom-up and top-down connections interplay through a complex network, with feedback inputs shape or modulate the processing of feed-forward visual information, e.g. attention-modulated visual processing [32]. Therefore, we speculate that the phase-shuffling of the movie eliminates the reliable response at higher visual areas, and results in the loss of reliable feedback control over early visual areas. Such feedback inputs likely serve as important drivers or modulators of reliable slowly fluctuating cortical responses observed with fMRI [33,34]. This interpretation is speculative, and awaits future studies to address in various spatial (network vs. synaptic levels) and temporal (seconds vs. milliseconds) scales with brain imaging and neural recording measurements.

The dense inter-connections between lower and higher visual areas makes it complicated to argue for any specific source of reliable activity during natural vision. The reliability of cortical responses at the lower and higher visual areas cannot be simply dissociated. The response reliability at higher visual areas requires their input areas to also exhibit reliable responses. Although our results highlight the essential role of natural content in driving reliable cortical fMRI activity, it does not imply that high-level visual features in natural movie stimuli account entirely for the intra- and inter-subject reproducible fMRI responses at any visual area. The scenario of hierarchical processing is necessary to support progressive and selective processing of various visual features underlying natural vision. While low-level visual features can be isolated from high-level features, it is not possible vice versa. More systematic understanding of the distributing visual processing should perhaps benefit from network analysis, as opposed to an over-simplified dichotomy of lower vs. higher level processing, or region-specific assignment of visual functions.

### Scene transitions contribute to fMRI activity and its reproducibility

With natural movie paradigms, previous studies emphasized the functional selectivity of reproducible activity at individual visual areas [5,6]. As supporting evidence for this functional selectivity, peak activity was retrospectively linked to the occurrence of specific objects or actions in the movie stimuli. Results from this study call for cautions in such analyses or interpretation. Scene transitions in almost all commercial movies occur as discrete events temporally superimposed with the continuous and complex movie stimulation. Previous studies also show that brain activity is, at least in part, time-locked to discrete transition events in movies [14], and such events cause a greater degree and extent of inter-subject correlation (ISC) in movie-stimulus evoked activity than those with single-shot, unstructured movies containing no scene transitions [3]. Therefore, it is likely of concern that scene transition events may evoke event-related responses in activity time series, to confound or mislead the functional interpretation of regional activity.

Using the regression based analysis, we found that scene transitions contributed to fMRI activity at extended cortical areas, most notably along the ventral pathway, e.g. V4 and ventral occipital (VO) areas, and less notably along the dorsal pathway, e.g. V3A/B, V7, intra-parietal sulcus (IPS) (Fig 5C). Moreover, the transition related activity partly accounted for the observed reproducibility of fMRI activity at these areas during natural movie stimulation (Fig 5D).

Since scene transitions contribute to fMRI activity and may confound the reproducibility of activity along the ventral visual stream, which is widely thought to be responsible for object recognition, one should perhaps be cautious interpreting activity time series at ventral visual areas. For example, the lateral-occipital complex (LOC) plays an important role in human object and face recognition and has been shown to respond more strongly to pictures of objects than to their scrambled images [35,36]. The ventral occipital-temporal cortex (VOT) consists of the fusiform face area (FFA) and the collateral sulcus (CoS) that are thought to be responsible for face and building recognition, respectively. The peak activity at these areas may indicate the occurrence of their encoded objects, or likely result from scene transitions. These two alternative possibilities should be considered for the interpretation of previous and future natural vision experiments.

These two possibilities are likely mixed but not mutually exclusive. Russ et al. showed that scene cuts (or transitions) did not account for significant variance in the fMRI signal [2]. Huth et al., showed that semantic information was encoded in the fMRI voxel time series at the ventral visual areas, even after regressing out the motion energy, which likely included scene transitions [37]. Therefore, it is plausible to retrieve rich semantic information from fMRI signals during movie watching, despite the confounding effects from scene transitions.

### Effects of gaze behavior on fMRI activity and reproducibility

In response to an identical movie stimulus, the gaze behavior may vary within and across subjects. As the retinotopic mapping is with respect to the center of the visual field defined by the gaze position, we expected the varying gaze behavior to affect the fMRI response at all or most retinotopic areas, as well as those areas involved in eye movement. In line with our expectation, the gaze behavior, described as the time-varying saccade amplitude, affected widely-spread brain regions including retinotopic areas as peripheral V1 and LGN, and specific regions in the oculomotor network including superior colliculus, parietal eye fields, frontal eye fields, supplementary eye fields and dorsal-lateral prefrontal cortex (Fig 5A), which are all related to controlling eye-movement and/or attention, as previously reported [38].

However, to our surprise, neither mathematically removing our gaze variation from fMRI time series nor experimentally controlling the subject’s eye movements (with the fixation) showed dramatic and wide-spread reduction in intra-subject correlation (Fig 4B and 4C). The gaze effects only influenced mainly in peripheral V1 with the mathematical regression and influenced a limited set of regions including peripheral part of V1, V3 and IPS with the fixation control. The limited gaze-related effects were perhaps partially attributable to the sub-optimal model used to describe the relationship between gaze movement and the fMRI signal. As this relationship was not fully understood and could likely be better described by other linear or non-linear models, the gaze-related effects may be under-estimated. Since we did not explore all possible models, we cannot rule out the possibility that eye movement might have stronger effects than were observed and reported in this paper. Despite this limitation in modeling, the contrast between free viewing and fixation conditions also revealed limited effects of the varying gaze behavior.
